# Predicting coercion during the course of psychiatric hospitalizations

**DOI:** 10.1192/j.eurpsy.2023.3

**Published:** 2023-01-26

**Authors:** Mario Müller, Nathalie Brackmann, Matthias Jäger, Anastasia Theodoridou, Stefan Vetter, Erich Seifritz, Florian Hotzy

**Affiliations:** 1 Department of Psychiatry, Psychotherapy and Psychosomatics, Psychiatric University Hospital Zurich, University of Zurich, Zürich, Switzerland; 2 Department of Forensic Psychiatry, Psychiatric University Hospital Zurich, University of Zurich, Zürich, Switzerland

**Keywords:** Coercion, forced medication, psychiatry, restraint, seclusion

## Abstract

**Background:**

Coercive measures (such as seclusion, mechanical restraint, and forced medication) during psychiatric inpatient treatment should be avoided whenever possible. Different interventions were already developed to reduce coercion, but for their effective application, it is crucial to know the risk factors of individuals and clinical situations that might be associated with coercion. Since the results of previous studies differ considerably the current study aims to fill this gap by evaluating the course of the exertion of coercion in detail.

**Methods:**

In this study, we analyzed clinical, procedural, and sociodemographic data from patients (*n* = 16,607 cases) who were treated as inpatients in Switzerland’s largest psychiatric institution with 320 beds during the years 2017 to 2020. We used regression models to identify predictors for the exertion of coercion, the number of coercive measures during a treatment episode and time until exertion of the first and last coercive measure.

**Results:**

Coercive measures are mostly used during the first days of treatment. We identified clinical parameters such as manic or psychotic episodes to be the most relevant predictors for the exertion of coercion. Cases with those disorders also received coercion more often and earlier in their treatment course than other diagnostic groups. Other promoting factors for frequency and early application of coercion were involuntary admission and factors of chronicity and clinical severity.

**Conclusions:**

Knowing the risk factors may help to target preventive strategies for those at highest risk. In particular, interventions should focus on the critical timeframe at the beginning of treatment.

## Introduction

The exertion of coercion during psychiatric treatment (seclusion, restraint, forced medication) is problematic and should be the last option in a cascade of less restrictive alternatives [1]. Most patients who experienced coercion describe feelings of helplessness [2, 3], fear [4], anger [5], humiliation [6], and a worsening of the therapeutic relationship [7] during and after the use of the coercive measure. Due to that, some patients refuse treatment in a future crisis [8], are skeptical or even hostile toward psychiatry [9], which is a risk to receive coercion again [10].

Nevertheless, some patients retrospectively state that they needed coercive measures [3] and that they want to be forced into treatment again, in the case of a future crisis [11]. Therefore, some patients see an intended “help” of a coercive measure, although this does not mean that they consider coercion as justified or helpful, especially not in the situation itself.

Although most mental healthcare professionals (HCP) view coercion as a violation against the fundamental right for freedom, most state that in some situations coercion is unavoidable [12–14] and that it is even beneficial for patients. Nevertheless, most HCP have a rather critical view on coercion and describe that its exertion is accompanied by feelings such as helplessness or guilt [15].

Due to the serious interference with personal rights to freedom and potential negative effects, it is a common aim to reduce coercion and therefore, foster preventive strategies. Nevertheless, to be able to apply preventive strategies in a targeted manner it is important to know which patients are at risk for coercive measures.

Previous studies showed that younger age [16, 17], female [16, 18] but also male gender [19], psychotic [20–26], bipolar [25, 27], personality [16, 28] and substance-use-related disorders [29], mental retardation [16], and higher symptomatology [20, 30, 31] were significantly associated with the exertion of coercion.

Besides, also procedural aspects like a history of former admissions [17, 29, 32], long durations of hospitalization [17, 28, 33], involuntary admission (IA) [20, 22, 24–27, 31, 34], and aggressive behavior prior to admission [20] were associated with a higher risk for coercion.

Nevertheless, there are differences between study sites (e.g., general vs. forensic treatment [35]), and previous analyses lack in information on the clinical course of patients who receive coercion. Only a few studies analyzed the timeframe in which coercion was applied. To our knowledge, no study searched in detail for differences in the duration until the first and last coercive measure was used regarding patient-related characteristics.

Based on this background, this study aimed to analyze predictors for the exertion of coercion. In particular, we were interested if risk factors differ during the course of treatment and if patients with psychosis are at higher risk to experience coercion during a longer period throughout inpatient treatment compared to persons with other diagnoses.

## Methods

### Sample and procedure

In total, the study sample consisted of *N* = 16,607 inpatient treatment episodes (cases) of 8,700 adult patients, which were carried out between January 1, 2017, to December 31, 2020 (full census) at the wards for adult treatment (320 beds) at the Psychiatric University Hospital Zurich, which is Switzerland’s largest psychiatric institution. It has a public service obligation and therefore treats patients with all psychiatric diagnoses and also receives patients with IA. Within the 4-year period, *n* = 3,760 (22.6%) cases of *n* = 2,656 patients were involuntarily admitted and *n* = 12,847 (77.4%) cases of *n* = 7,093 patients were based on a voluntary status. The sample consisted of slightly more males (*n* = 9,294, 56.0%) than females. Age ranged between 18 and 65 years (mean age = 39.9 years; SD = 12.3 years). The majority was single, had a Swiss nationality, had sufficient German language skills, and admission was mostly initiated by self-referrals. For further details on sociodemographic characteristics as well as on clinical variables please refer to Table 1.

**Table 1.** Distributions of coercive measures and associations with sociodemographic and clinical characteristics.

		Coercion			
	Total	No	Yes	*p*-value^a^	Adj. OR (95%CI)^b^
Gender				**0.036**	
Female (col%)	7,313 (44.0)	6,845 (44.3)	468 (41.1)		0.95 (0.80–1.13)
Age in years (M ± SD)	39.9 ± 12.3	39.9 ± 12.3	39.9 ± 12.5	0.952	0.92 (0.84–1.02)
Civil status (col%)				**<0.001** a,c > b e > a,b,d	
a. Single	10,002 (60.3)	9,298 (60.2)	704 (61.8)		Ref.
b. Married	2,494 (15.0)	2,369 (15.3)	125 (11.0)		0.82 (0.62–1.09)
c. Widowed	129 (0.8)	117 (0.8)	12 (1.1)		1.21 (0.49–3.01)
d. Divorced	2,889 (17.4)	2,713 (17.6)	176 (15.4)		1.02 (0.78–1.32)
e. Unknown	1,084 (6.5)	961 (6.2)	123 (10.8)		**1.44 (1.06**–1.95)*
Residential status (col%)				**<0.001** a,c,d,e > b e > a	
a. Swiss	11.830 (72.1)	11,016 (72.1)	814 (72.8)		Ref.
b. Official permit	3,261 (19.9)	3,084 (20.2)	177 (15.8)		0.85 (0.67–1.07)
c. Asylum seeker	367 (2.2)	336 (2.2)	31 (2.8)		1.13 (0.64–1.98)
d. Tourist	133 (0.8)	120 (0.8)	13 (1.2)		0.72 (0.28–1.82)
e. Other	812 (5.0)	729 (4.8)	83 (7.4)		1.25 (0.89–1.77)
German language skills (col%)				**<0.001** b > a	
a. Well	14,453 (87.9)	13,515 (88.2)	938 (83.5)		Ref.
b. Difficult	1,483 (9.0)	1,339 (8.7)	144 (12.8)		1.32 (1.00–1.74)
c. Interpreter required	512 (3.1)	471 (3.1)	41 (3.7)		1.08 (0.67–1.75)
Diagnostic category (col%)				**<0.001** a > b,d,f,g b > d,f c > b,d,f,g,h e > a,b,c,d,f,g,h g > b,d,f h > b,d,f,g	
a. Organic	265 (1.6)	226 (1.5)	39 (3.4)		**2.52 (1.50**–4.22)***
b. Substance	4,636 (27.9)	4,508 (29.2)	128 (11.2)		Ref.
c. Psychotic	4,006 (24.1)	3,360 (21.7)	646 (56.7)		**3.67 (2.85**–4.74)***
d. Depressive	3,560 (21.4)	3,513 (22.7)	47 (4.1)		**0.61 (0.40**–0.91)*
e. Manic	387 (2.3)	253 (1.6)	134 (11.8)		**11.47 (7.96**–16.54)***
f. Neurotic	2,081 (12.5)	2,042 (13.2)	39 (3.4)		0.79 (0.48–1.30)
g. Personality	1,383 (8.3)	1,306 (8.4)	77 (6.8)		**2.11 (1.43**–3.10)***
h. Others	289 (1.7)	259 (1.7)	30 (2.6)		1.54 (0.77–3.08)
Involuntary admission					
Yes (col%)	3,760 (22.6)	2,886 (18.7)	874 (76.7)	**<0.001**	**6.04 (4.77**–7.64)***
Initiant of hospitalization (col%)				**<0.001** a > b,d,e,f,g b > f,g c > ALL d > b,e,f,g e > f,g	
a. GP	1,583 (9.5)	1,343 (8.7)	240 (21.1)		**2.37 (1.63**–3.45)***
b. Psychiatrist	4,077 (24.6)	3,799 (24.6)	278 (24.5)		**1.55 (1.11**–2.16)*
c. Emergency physician	1,002 (6.0)	711 (4.6)	291 (25.6)		**2.41 (1.63**–3.56)***
d. Somatic clinic	2,230 (13.4)	2,032 (13.1)	198 (17.4)		1.36 (0.92–2.00)
e. Authorities, others	901 (5.4)	855 (5.5)	46 (4.1)		1.12 (0.65–1.91)
f. Patient	6,271 (37.8)	6,197 (40.1)	74 (6.5)		Ref.
g. Relatives	535 (3.2)	525 (3.4)	10 (0.9)		1.13 (0.54–2.35)
Number of previous hospitalizations (M ± SD)	6.5 ± 10.4	6.4 ± 10.2	8.5 ± 12.3	**<0.001**	**1.11 (1.03**–1.20)**
Duration of hospitalization (days) (M ± SD)	24.6 ± 26.8	23.6 ± 25.4	38.2 ± 38.3	**<0.001**	**1.37 (1.29**–1.46)***
GAF at admission (M ± SD)	39.2 ± 13.8	39.9 ± 13.5	29.3 ± 13.0	**<0.001**	**0.71 (0.65**–79)***
GAF at discharge (M ± SD)	54.1 ± 15.3	54.4 ± 15.1	50.7 ± 17.2	**<0.001**	0.97 (0.89–1.06)
HoNOS total admission score (M ± SD)	24.1 ± 12.2	23.8 ± 12.2	28.6 ± 12.0	**<0.001**	**1.30 (1.16**–1.45)***
HoNOS total discharge score (M ± SD)	19.0 ± 9.9	18.7 ± 9.7	21.7 ± 11.8	**<0.001**	1.05 (0.95–1.17)

*Note:* Numbers refer to case-wise counts;

a Posthoc pairwise comparisons were given for overall significant models;

b Continuous variables were transformed into z-scores prior to logistic regression modelling;

M±SD = Mean ± Standard deviation; col% = Column %; OR = Odds ratio; 95%CI = 95% Confidence interval; Ref. = Reference category; * p < .05; ** p < .01; *** p < .001; Significant values are print in bold; GP = General practioner; GAF = General Assessment of Functioning; HoNOS = Health of the Nation Outcome Scale.

All analyses and numbers reported in this study refer to case-wise counts.

We used data of the patients’ routine documentation and quality assessment, which were rated by the responsible clinicians. Clinicians were obliged by the Swiss Department of Health to complete all considered study measures at admission and discharge as part of the routine clinical care procedure.

#### Ethics

The study was reviewed and approved by the Cantonal Ethics Commission of Zurich, Switzerland (Reference Number EK: Req-2019-02470). It was performed in accordance with the ethical standards laid down in the 1964 Declaration of Helsinki and its later amendments. An identification of the patients was not possible at any time of the study.

### Coercive measures

The coercive measures [36] which were included in the analysis and referred to as “coercion” in this article were:

Seclusion: Locking the patient in a single room with surveillance through a window with an interval of 15 min [19].

Mechanical restraint: Strapping the patient to a bed with mechanical devices (belts). At the present psychiatric institution, bed belts with 5-point restraints (arms, legs, and torso) or less are used if necessary. This measure is always continuously accompanied by HCP [19].

Forced medication: Applying medication (typically tranquilizers or antipsychotics) against the patients will orally or as an intramuscular injection [19].

### Raters and training

Raters were either senior psychiatrists, psychiatric residents, or clinical psychologists. All relevant information was derived directly by standardized exploration interviews and behavioral observation, as well as indirectly by reports from nursing staff, social workers, and significant others.

All raters were trained in specific workshops on clinical assessment as well as on the use and objectives of clinical measures, such as the Health of the Nation Outcome Scales (HoNOS [37]) for rating clinical presentation and the Global Assessment of Functioning (GAF [38]) for assessing functional levels. The training followed a standardized schedule, with case vignettes or patient videos as examples. Refresher training was provided on a regular basis.

### Measurements

#### Global Assessment of Functioning

The GAF is a 100-point single-item observer-rated scale. It rates overall functioning on a continuum from mental health to mental illness and should reflect the past 7 days [38]. The scale ranges from 1 (representing the most impaired individual) to 100 (representing the healthiest individual), 0 denoting insufficient information to make a clinical judgment.

#### Health of the Nation Outcome Scales

The HoNOS is an observer-rated scale and consists of 12 items with a 5-point Likert scale response format from 0 (no problems) to 4 (severe/very severe problems), scores above 2 are considered clinically significant [37]. These items address dimensions of aggressiveness, nonaccidental self-injury, problem drinking or drug taking, cognitive problems, physical illness or disability problems, hallucinations and delusions, depressed mood, other mental and behavioral problems, problems with relationships, problems with activities of daily living, problems with living conditions, and problems with occupation and activities. We evaluated the HoNOS on total score level in case of at least nine responses.

#### Diagnostic assessments

Patients were diagnosed according to the International Classification of Diseases, 10th edition (ICD-10 [39]) criteria. For the purpose of the current study, the sample was divided into the following subgroups according to the patients’ primary diagnosis, namely [1] mental disorders due to known physiological disorders (organic disorders); [2] substance use disorders (substance abuse); [3] schizophrenia and other psychotic disorders (psychotic episodes); [4] affective disorders; except manic episodes (depressive episodes); [5] manic episodes and bipolar disorders; except depressive or mixed episodes (manic episodes); [6] anxiety and somatoform disorders (neurotic disorders); [7] personality disorders (personality disorders); and [8] remaining diagnoses (others).

#### Statistical methods

Bivariate associations between coercion and sociodemographic and clinical variables were calculated. Frequencies and percentages are given for categorical and means (M) and standard deviations (SD) for continuous variables. To examine differences between conditions (coercion—yes/no) we calculated Chi-square statistics for categorical as well as one-way ANOVAs for continuous variables with Bonferroni post hoc comparisons for pairwise differences. Pairwise group comparisons for categorical data were performed using multinomial logistic regressions with changing reference categories. Finally, a multivariate binary logistic regression model was fitted to estimate the association of coercion with previously determined predictor candidates except for gender and age, which were included anyway. Odds ratios (OR) were calculated with 95% confidence interval (95%CI) with no coercion serving as the reference condition. Categorical predictors with more than two categories were treated in a logical sense, that is, the largest category was used as a reference for final modeling.

In addition, we compared the number of coercive measures as well as the mean time difference from admission to the first and last coercive measure, respectively, with regard to the same set of variables.

For bivariate analyses, mean time differences were compared in the same manner as described above, while associations with continuous variables were estimated with simple Pearson Product-Moment correlations. For both outcomes separately, that is, time to first and time to last coercive measure, significant predictor candidates were fitted in multivariate linear regression models. Consistent with previous modeling, gender and age were treated as standard covariates irrespective of whether they linked to outcomes in bivariate analyses, and reference categories of categorical predictors were chosen by their largest level [40]. Models examining time differences were conducted using cases with coercion only.

Separate analyses of the different exerted measures (seclusion, mechanical restraint, and forced medication) yielded similar patterns of results, that is, no different risk factors for specific measures were found. Therefore, we combined the measures and used one variable “coercion” as outcome.

All analyses were conducted using STATA/SE 16 (StataCorp. 2019. *Stata Statistical Software: Release 16.* College Station, TX: StataCorp LLC).

## Results

### Descriptive analyses

Seclusion was exerted in 6.0% (*n* = 1,002), mechanical restraint in 0.3% (*n* = 44), and forced medication in 4.0% (*n* = 662) of all cases. In total, in 6.9% (*n* = 1,140) of the included cases coercion was exerted. In 795 of all cases with coercion (69.7%) at least a second coercive measure was exerted.

Table 1 displays the associations of coercion with sociodemographic and clinical variables. Accordingly, exertion of coercion was bi-variately linked to male gender, to civil and residential status (for more details see Table 1), as well as to insufficient language skills. Moreover, patients with psychotic and manic episodes, those referred to hospitalization primarily by a general practitioner or emergency physician as well as those with IA were more likely to experience coercion during treatment. Moreover, the exertion of coercion was associated with lower clinical functioning and clinical presentation at both admission and discharge. Functional improvement during treatment was significantly higher in patients who experienced coercion than in those without (GAF difference score: 21.1 ± 19.3 vs. 14.3 ± 15.9; *p* < 0.001; not tabulated). Similarly, those with coercion had a higher improvement of clinical presentation during treatment (HoNOS difference score: −6.9 ± 13.1 vs. 4.5 ± 8.9; *p* < 0.001). Other indicators of clinical severity and chronicity, such as the length of stay and/or the number of previous hospitalizations were also positively linked to coercion. More details are presented in Table 1.

After adjusting for all bi-variately associated variables (plus age) in a final model manic episodes were most likely to be linked to coercion, followed by psychotic episodes and organic as well as personality disorders, while depressive episodes were negatively linked to coercion. Other factors that increased the likelihood for coercion in the adjusted model were a history of previous hospitalization, IA, longer duration of hospitalization as well as clinical severity (lower functioning and clinical presentation) at time of admission. Finally, compared to self-referrals by the patient itself referrals by a general or emergency physician or psychiatrist were linked to higher odds of coercion.

Table 2 displays descriptive statistics of the overall number of coercive measures for the total study sample as well as their associations and differences regarding potentially explaining variables. Overall, in our sample, the mean number of measures was 0.62 (SD = 5.25; range 0–183). Bivariate analyses revealed a higher number of coercive measures to be linked to higher age, being widowed, a psychotic or manic episode, IA, initiant of hospitalization (especially referrals by an emergency physician or general practitioner), frequent previous and longer treatment duration, lower clinical functioning (at admission and discharge) and more impairment regarding clinical presentation at time of admission.

**Table 2.** Frequency of coercive measures and associations with sociodemographic and clinical characteristics.

	M ± SD/correlation coefficient	*p*-value^a^	Adj. model (beta weight)
Gender		0.427	
Male	0.65 ± 5.47		Ref.
Female	0.58 ± 4.95		0.00
Age in years	0.02	**0.006**	0.01
Civil status		**<0.001** c > ALL	
a) Single	0.68 ± 5.74		Ref.
b) Married	0.38 ± 3.11		−0.02*
c) Widowed	2.87 ± 17.87		0.03***
d) Divorced	0.47 ± 3.70		−0.02*
e) Unknown	0.77 ± 4.49		0.00
Residential status		**0.004** e > b	
a) Swiss	0.64 ± 5.34		Ref.
b) Official permit	0.36 ± 3.53		**−**0.01
c) Asylum seeker	1.03 ± 10.12		0.01
d) Tourist	0.22 ± 0.85		**−**0.02
e) Other	0.98 ± 6.11		0.00
German language skills		0.079	
a) Well	0.60 ± 5.15		—
b) Difficult	0.83 ± 6.42		—
c) Interpreter required	0.26 ± 1.87		—
Diagnostic category		**<0.001** c > b,d,f,g e > ALL	
a) Organic	0.98 ± 4.46		0.00
b) Substance	0.07 ± 0.88		Ref.
c) Psychotic	1.90 ± 9.61		0.10***
d) Depressive	0.06 ± 1.44		0.00
e) Manic	3.07 ± 10.61		0.06***
f) Neurotic	0.05 ± 0.44		0.02
g) Personality	0.23 ± 2.20		0.01
h) Others	0.93 ± 5.27		−0.01
Involuntary admission			
No	0.12 ± 1.96	**<0.001** Yes > No	Ref.
Yes	2.32 ± 10.23		0.13***
Initiant of hospitalization		**<0.001** a > b,d,e,f,g b > f c > ALL d > f	
a) GP	1.45 ± 7.80		0.01
b) Psychiatrist	0.73 ± 6.03		−0.01
c) Emergency physician	2.94 ± 11.81		0.02*
d) Somatic clinic	0.61 ± 4.41		−0.04***
e) Authorities, others	0.31 ± 2.77		−0.01
f) Patient	0.04 ± 0.76		Ref.
g) Relatives	0.32 ± 4.08		0.00
Number of previous hospitalizations	0.02	**0.022**	0.00
Duration of hospitalization (days)	0.19	**<0.001**	0.18***
GAF at admission	−0.12	**<0.001**	−0.05***
GAF at discharge	−0.02	**0.003**	0.01
HoNOS total admission score	0.04	**<0.001**	0.01
HoNOS total discharge score	0.01	0.511	—

*Note:* Numbers refer to case-wise counts;

a Posthoc pairwise comparisons were given for overall significant models;

M±SD = Mean ± Standard deviation; Ref. = Reference category; * p < .05; *** p < .001; Significant values are print in bold; GP = General practioner; GAF = General Assessment of Functioning; HoNOS = Health of the Nation Outcome Scale.

After adjusting for all significantly linked variables (plus gender) being widowed, having psychotic or manic episodes, IA, referrals by an emergency physician, longer treatment durations and lower baseline functioning remained significant predictors for higher numbers of coercive measures. In contrast, those who were married or divorced or had been referred by a somatic hospital had significantly lower numbers of coercion.

### Clinical course regarding the exertion of coercion

In cases that experienced at least one coercive measure, the mean number of coercive measures within one stay was 9.0 (SD = 18.0). In those cases, the mean duration until the first measure was exerted was 7.68 days (SD = 12.97). In detail, 25% had their first coercive measure at the first day of hospitalization, 50% within 3 days and 75% within 8 days after admission.

In 795 of all cases with coercion (69.7%) at least a second coercive measure was exerted. From those with two or more measures, the mean duration until the last measure was exerted was 21.98 days (SD = 27.68). In 25% the last measure was exerted until the third day of stay. In 50%, no more coercion was exerted later than 11 days and in 75% none after 32 days of hospitalization. Table 3 displays associations of predictor variables with time to first and last coercion for those patients with coercion during their treatment.

**Table 3.** Bivariate and multivariate associations between sociodemographic and clinical factors and time from admission to first/last coercive measure in the subsample of patients with coercive measures.

	Time to first coercive measure			Time to last coercive measure		
	M ± SD/correlation coefficient	*p*-value^a^	Adj. model (beta weight)	M ± SD/correlation coefficient	*p*-value^a^	Adj. model—(beta weight)
Gender		0.189			0.908	
Male	7.26 ± 12.37		Ref.	21.88 ± 29.69		Ref.
Female	8.28 ± 13.77		−0.02	22.11 ± 24.52		−0.02
Age in years	−0.01	0.841	0.02	0.03	0.421	0.03
Civil status		**0.023**			**<0.001** a > b,d,e c > b,e	
a) Single	8.55 ± 14.29		Ref.	25.71 ± 31.43		Ref.
b) Married	5.79 ± 8.91		−0.04	15.33 ± 18.27		−0.03
c) Widowed	4.17 ± 5.04		−0.04	42.33 ± 50.31		0.04
d) Divorced	7.51 ± 12.73		−0.01	17.27 ± 17.61		−0.06*
e) Unknown	5.18 ± 7.79		−0.01	13.20 ± 15.48		0.00
Residential status		0.850			0.626	
a) Swiss	7.61 ± 12.53		—	22.72 ± 29.51		
b) Official permit	8.24 ± 15.11		—	20.04 ± 22.58		—
c) Asylum seeker	5.94 ± 9.69		—	18.35 ± 24.87		—
d) Tourist	6.31 ± 7.90		—	10.75 ± 8.76		—
e) Other	6.94 ± 8.20		—	22.02 ± 22.45		—
German language skills		0.552			0.080	
a) Well	7.60 ± 12.16		—	22.60 ± 28.59		—
b) Difficult	7.01 ± 10.92		—	20.85 ± 24.07		—
c) Interpreter required	9.44 ± 23.24		—	9.83 ± 13.36		—
Diagnostic category		**<0.001** b,c,d,g > e			**0.007**	
a) Organic	10.18 ± 19.62		−0.03	27.12 ± 32.30		0.00
b) Substance	8.02 ± 11.67		Ref.	14.22 ± 22.23		Ref.
c) Psychotic	7.22 ± 12.11		−0.13**	24.45 ± 29.13		−0.03
d) Depressive	15.23 ± 17.25		0.03	24.96 ± 23.98		−0.04
e) Manic	3.50 ± 4.83		−0.15***	15.61 ± 25.86		−0.04
f) Neurotic	6.95 ± 12.57		−0.02	11.84 ± 16.23		−0.03
g) Personality	11.68 ± 19.08		0.04	21.97 ± 26.04		0.01
h) Others	10.17 ± 16.03		0.04	23.68 ± 17.18		0.02
Involuntary admission		**<0.001** No > Yes			0.443	
No	11.06 ± 16.55		Ref.	23.46 ± 31.93		—
Yes	6.64 ± 11.46		−0.03	21.59 ± 26.48		—
Initiant of hospitalization		**<0.001** f > a,b,c,d,e			**0.023** f > d	
a) GP	6.49 ± 9.10		−0.22***	23.08 ± 30.42		−0.12*
b) Psychiatrist	8.44 ± 12.45		−0.21***	24.37 ± 24.58		−0.13*
c) Emergency physician	6.12 ± 11.86		−0.21***	20.31 ± 25.31		−0.13*
d) Somatic clinic	6.86 ± 13.26		−0.20***	16.73 ± 22.18		−0.12**
e) Authorities, others	7.54 ± 14.01		−0.10**	16.25 ± 23.78		−0.07*
f) Patient	16.86 ± 21.92		Ref.	33.42 ± 50.58		Ref.
g) Relatives	6.80 ± 8.00		−0.05	23.29 ± 16.43		−0.02
Number of previous hospitalizations	0.06	0.054	—	0.12	**0.001**	0.09**
Duration of hospitalization (days)	0.46	**<0.001**	0.45***	0.76	**<0.001**	0.75***
GAF at admission	0.12	**<0.001**	0.09**	0.02	0.606	—
GAF at discharge	−0.06	0.057	—	−0.04	0.259	—
HoNOS total admission score	0.01	0.807	—	0.01	0.767	—
HoNOS total discharge score	0.02	0.574	—	−0.03	0.455	—

*Note:* Numbers refer to case-wise counts;

a Posthoc pairwise comparisons were given for overall significant models;

M±SD = Mean ± Standard deviation; Ref. = Reference category; * p < .05; ** p < .01; *** p < .001; Significant values are print in bold; GP = General practioner; GAF = General Assessment of Functioning; HoNOS = Health of the Nation Outcome Scale.

A shorter time to the first measure was bi-variately linked to manic or psychotic episodes, IA, to all nonself-referrals, a shorter duration of hospitalization as well as lower clinical functioning at time of admission.

After adjusting for all predictor candidates (plus gender and age) manic and psychotic episodes and referrals by others (than self-referrals), shorter duration of hospitalization and lower clinical functioning at admission remained significant predictors for a shorter time to first coercion.

In case of two or more measures, a longer time until the last coercive measure was associated with being single or widowed, patients’ self-referrals, a higher number of previous hospitalizations and longer durations of inpatient treatment. In the adjusted model, patients’ self-referrals were linked to a significantly longer duration until the last coercive measure was used. In contrast, being divorced, having less previous admissions and shorter durations of inpatient treatment remained significant predictors for a shorter duration until the last coercive measure.

## Discussion

In this study, we analyzed predictors for the exertion of coercion during psychiatric inpatient treatment. Predictors with the highest risk for coercion were manic episodes, psychotic disorders and IA.

We further assessed the frequency of measures per predictor and as far as we know, this is the first study to analyze factors which are associated with an earlier and prolonged exertion of coercion. We found that 50% of those who experienced coercion were affected within the first 3 days of treatment, whereas only 25% experienced their first coercive measure after 8 days. Also, within 8 days 50% had experienced their last measure.

### Clinical predictors and possible strategies to reduce coercion

Regarding diagnostic categories, we found manic and psychotic episodes, organic and personality disorders in descending order to be associated with an increased risk for coercion. Besides IA, manic and psychotic episodes were the variables with the highest likelihood for coercion. Patients with those disorders and those with lower clinical functioning also had a significantly higher risk for early exertion of coercive measures, whereas the time until the last measure was exerted was not significantly associated with clinical parameters. Our findings are in line with previous studies describing psychotic and manic/bipolar diagnoses [20–26] and higher clinical symptom severity [20, 30, 31] to be important risk factors for the exertion of coercion. In agreement with previous studies [25, 27, 30], affective (others than manic) disorders were negatively linked to coercion.

Confirming previous studies, IA was a prominent risk factor for the exertion of coercion [20, 22, 24–27, 31, 34]. Although Switzerland does not rely on the “danger criterion” in its regulation of IA [41], danger to others or self-endangerment caused by a psychiatric condition are the most common reasons for IA [42]. An IA in itself is of coercive nature and may be perceived by patients as such. This in turn may aggravate destructive behavior and therefore increase the risk for coercion. Besides that, severe clinical impairment, delusional symptoms, poor impulse control, or lack of insight can increase the risk for aggressive behavior and for coercion [30, 43–45].

Furthermore, an admission for inpatient treatment requires changes in daily routines and an adaption to other patients and HCP. This demands adjustment skills from the patient’s side. Waiting times on the ward, not being allowed to leave and rigid ward rules can overstrain the capacity to adapt and then lead to aggression [45]. HCP should aim to adapt to each patient’s needs in a way that on one side enables a treatment process and on the other side strengthens the perceived autonomy [46]. On the ward level, the room size, ward atmosphere, and the support in the use of skills to reduce stress were shown to reduce coercion [47].

The drastic reduction of coercion after the first days of treatment in our study might indicate that treatment efforts successfully reduce aggressive behavior toward oneself or others. Nevertheless, during the first days of treatment, HCP are also less familiar with new patients which hardens anticipation of their needs and therefore, might result in escalation and the exertion of coercion more often [21]. The active use of risk-assessment tools right after admission and on a regularly basis can help to identify those at risk for aggressive behavior and coercion [48]. Advance directives might be helpful as the patients can predefine what strategies were helpful in the past [49].

### Organizational predictors and possible strategies to reduce coercion

In line with other studies, we also identified other risk factors, such as a history of hospitalizations [25]. We also found that shorter hospitalizations are associated with a higher risk for earlier exertion of coercion. Due to a lack of other options, some aggressive persons might be admitted to acute wards for purposes of behavior control [50] and due to their aggressive behavior, be at risk for coercion. If diagnostic processes reveal that no treatment is needed a fast discharge might be initiated. This implies the importance to keep an eye on the admission process. From the referring physicians perspective, the question if IA is needed or not is sometimes challenging, especially if physicians are insecure with the criteria for IA and psychiatric disorders in general [51]. We found a higher risk for coercion in patients referred by GP’s, emergency physicians, and psychiatrists. In the state of Zurich, these physicians are involved in the emergency service system and account for a relevant number of IA. Interestingly, although hospital physicians are responsible for a relevant percentage of IA [42], referrals of this group were not associated with an increased risk for coercion. Moreover, patients referred by hospital physicians had a significantly lower risk for frequent coercive measures. On the other hand, when a patient was referred by emergency physicians, the risk for frequent coercive measures was significantly increased. This implicates that the patient groups referred by different physicians differ regarding their risk to experience coercion, irrespective of their admission status (voluntarily or involuntarily).

Previous studies showed that an asylum-seeking status was associated with a higher risk for coercion and IA [52–54]. In our models, the residential status was not significantly associated with a risk for coercion. Although nearly a fifth of the treated patients had language difficulties, this did not go ahead with an increased risk for coercion as it was described in previous studies [55]. Skills of HCP, but also the patients themselves to use nonverbal communication skills and de-escalation interventions other than talking are important resources to avoid an increased risk for coercion in this group.

### Limitations

This study was performed only at one clinic in Switzerland. However, the clinic is Switzerland’s largest psychiatric institution with a public service obligation, which makes its patient sample highly comparable to those of other clinics with a public service obligation.

Another limitation refers to the data for this study, which are based on clinical routine data and quality assessment parameters. However, even if generalization of our results has to be done with caution [56] there is evidence that diagnostic accuracy for most common mental health disorders in clinical routine is rather high [57]. Moreover, the validity of the routine data for the measurements used in this study was proven several times in previous research [58]. The study design did not allow to assess the respective reasons for the exertion of coercion, nor was it possible to assess the subjective perception of the coercive measures in the affected patients.

Besides these limitations, some strengths of this study are the large number of included patients and the duration of the analyzed period.

## Conclusion

We could show that besides some clinical and nonclinical characteristics of patients, also the administrative way of referral is a relevant predictor for the risk for coercion during inpatient treatment. Referrals by general or emergency physicians were most frequently linked to coercive measures. Also, we showed that patients with manic or psychotic episodes experience their first/last coercive measure earlier than others. As most coercive measures happen to be exerted during the first week of treatment, high efforts should be made to use strategies such as interventions to strengthen the patients autonomy [46], de-escalation techniques, good staff-patient ratio, leisure time services, and a calming ward atmosphere [47] during this period and especially in patients with risk factors.

## Data Availability

Data can be made available upon request and clarification of the purpose.

## References

[1] Chieze M, Clavien C, Kaiser S, Hurst S. Coercive measures in psychiatry: a review of ethical arguments. Front Psych. 2021;12:790886. doi:10.3389/fpsyt.2021.790886. PMC8712490 34970171

[2] Bergk J, Flammer E, Steinert T. “Coercion experience scale" (CES) - validation of a questionnaire on coercive measures. BMC Psychiatry. 2010;10:5. doi:10.1186/1471-244X-10-5. 20074355 PMC2837616

[3] Greenberg WM, Moore-Duncan L, Herron R. Patients’ attitudes toward having been forcibly medicated. Bull Am Acad Psychiatry Law. 1996;24(4):513–24. 9001749

[4] Naber D, Kircher T, Hessel K. Schizophrenic patients’ retrospective attitudes regarding involuntary psychopharmacological treatment and restraint. Eur Psychiatry. 1996;11(1):7–11. doi:10.1016/0924-9338(96)80452-4. 11660396

[5] Haglund K, Von Knorring L, Von Essen L. Forced medication in psychiatric care: patient experiences and nurse perceptions. J Psychiatr Ment Health Nurs. 2003;10(1):65–72. doi:10.1046/j.1365-2850.2003.00555.x. 12558923

[6] Whittington R, Bowers L, Nolan P, Simpson A, Neil L. Approval ratings of inpatient coercive interventions in a national sample of mental health service users and staff in England. Psychiatr Serv. 2009;60(6):792–8. doi:10.1176/ps.2009.60.6.792. 19487349

[7] Theodoridou A, Schlatter F, Ajdacic V, Roessler W, Jaeger M. Therapeutic relationship in the context of perceived coercion in a psychiatric population. Psychiatry Res. 2012;200(2–3):939–44. doi:10.1016/j.psychres.2012.04.012. 22575342

[8] Swartz MS, Swanson JW, Hannon MJ. Does fear of coercion keep people away from mental health treatment? Evidence from a survey of persons with schizophrenia and mental health professionals. Behav Sci Law. 2003;21(4):459–72. doi:10.1002/bsl.539. 12898502

[9] Mielau J, Altunbay J, Lehmann A, Bermpohl F, Heinz A, Montag C. The influence of coercive measures on patients’ stances towards psychiatric institutions. Int J Psychiatry Clin Pract. 2018;22(2):115–22. doi:10.1080/13651501.2017.1383437. 28978249

[10] Van der Post LF, Peen J, Visch I, Mulder CL, Beekman AT, Dekker JJ. Patient perspectives and the risk of compulsory admission: the Amsterdam study of acute psychiatry V. Int J Soc Psychiatry. 2014;60(2):125–33. doi:10.1177/0020764012470234. 23333906

[11] Lucksted A, Coursey RD. Consumer perceptions of pressure and force in psychiatric treatments. Psychiatr Serv. 1995;46(2):146–52. doi:10.1176/ps.46.2.146. 7712250

[12] Lepping P, Steinert T, Gebhardt RP, Roettgers HR. Attitudes of mental health professionals and lay-people towards involuntary admission and treatment in England and Germany - a questionnaire analysis. Eur Psychiatry. 2004;19(2):91–5. doi:10.1016/j.eurpsy.2003.11.001. 15051108

[13] Diseth RR, Bogwald KP, Hoglend PA. Attitudes among stakeholders towards compulsory mental health care in Norway. Int J Law Psychiatry. 2011;34(1):1–6. doi:10.1016/j.ijlp.2010.11.001. 21144587

[14] Morandi S, Silva B, Mendez Rubio M, Bonsack C, Golay P. Mental health professionals’ feelings and attitudes towards coercion. Int J Law Psychiatry. 2021;74:101665. doi:10.1016/j.ijlp.2020.101665. 33401095

[15] Krieger E, Moritz S, Lincoln TM, Fischer R, Nagel M. Coercion in psychiatry: a cross-sectional study on staff views and emotions. J Psychiatr Ment Health Nurs. 2021;28(2):149–62. doi:10.1111/jpm.12643. 32348607

[16] Beck NC, Durrett C, Stinson J, Coleman J, Stuve P, Menditto A. Trajectories of seclusion and restraint use at a state psychiatric hospital. Psychiatr Serv. 2008;59(9):1027–32. doi:10.1176/ps.2008.59.9.1027. 18757596

[17] Hendryx M, Trusevich Y, Coyle F, Short R, Roll J. The distribution and frequency of seclusion and/or restraint among psychiatric inpatients. J Behav Health Serv Res. 2010;37(2):272–81. doi:10.1007/s11414-009-9191-1. 19757076

[18] Sercan M, Bilici R. Restraint variables in a regional mental health hospital in Turkey. Turk J Psychiatry. 2009;20(1):37–48. 19306125

[19] Kalisova L, Raboch J, Nawka A, Sampogna G, Cihal L, Kallert TW, et al. Do patient and ward-related characteristics influence the use of coercive measures? Results from the EUNOMIA international study. Soc Psychiatry Psychiatr Epidemiol. 2014;49(10):1619–29. doi:10.1007/s00127-014-0872-6. 24737189

[20] Flammer E, Steinert T, Eisele F, Bergk J, Uhlmann C. Who is subjected to coercive measures as a psychiatric inpatient? A multi-level analysis. Clin Pract Epidemiol Ment Health. 2013;9:110–9. doi:10.2174/1745017901309010110. 23986786 PMC3744855

[21] Keski-Valkama A, Sailas E, Eronen M, Koivisto AM, Lonnqvist J, Kaltiala-Heino R. Who are the restrained and secluded patients: a 15-year nationwide study. Soc Psychiatry Psychiatr Epidemiol. 2010;45(11):1087–93. doi:10.1007/s00127-009-0150-1. 19844645

[22] Kaltiala-Heino R, Valimaki M, Korkeila J, Tuohimaki C, Lehtinen V. Involuntary medication in psychiatric inpatient treatment. Eur Psychiatry. 2003;18(6):290–5. doi:10.1016/j.eurpsy.2003.07.002. 14611924

[23] El-Badri SM, Mellsop G. A study of the use of seclusion in an acute psychiatric service. Aust N Z J Psychiatry. 2002;36(3):399–403. doi:10.1046/j.1440-1614.2002.01003.x. 12060190

[24] Odawara T, Narita H, Yamada Y, Fujita J, Yamada T, Hirayasu Y. Use of restraint in a general hospital psychiatric unit in Japan. Psychiatry Clin Neurosci. 2005;59(5):605–9. doi:10.1111/j.1440-1819.2005.01422.x. 16194266

[25] Knutzen M, Mjosund NH, Eidhammer G, Lorentzen S, Opjordsmoen S, Sandvik L, et al. Characteristics of psychiatric inpatients who experienced restraint and those who did not: a case-control study. Psychiatr Serv. 2011;62(5):492–7. doi:10.1176/ps.62.5.pss6205_0492. 21532074

[26] Georgieva I, Vesselinov R, Mulder CL. Early detection of risk factors for seclusion and restraint: a prospective study. Early Interv Psychiatry. 2012;6(4):415–22. doi:10.1111/j.1751-7893.2011.00330.x. 22277018

[27] Van de Sande R, Noorthoorn E, Wierdsma A, Hellendoorn E, van der Staak C, Mulder CL, et al. Association between short-term structured risk assessment outcomes and seclusion. Int J Ment Health Nurs. 2013;22(6):475–84. doi:10.1111/inm.12033. 23841809

[28] Knutzen M, Bjorkly S, Eidhammer G, Lorentzen S, Mjosund NH, Opjordsmoen S, et al. Characteristics of patients frequently subjected to pharmacological and mechanical restraint - a register study in three Norwegian acute psychiatric wards. Psychiatry Res. 2014;215(1):127–33. doi:10.1016/j.psychres.2013.10.024. 24230996

[29] Korkeila JA, Tuohimaki C, Kaltiala-Heino R, Lehtinen V, Joukamaa M. Predicting use of coercive measures in Finland. Nord J Psychiatry. 2002;56(5):339–45. doi:10.1080/080394802760322105. 12470307

[30] Lay B, Nordt C, Roessler W. Variation in use of coercive measures in psychiatric hospitals. Eur Psychiatry. 2011;26(4):244–51. doi:10.1016/j.eurpsy.2010.11.007. 21296560

[31] Dazzi F, Tarsitani L, Di Nunzio M, Trincia V, Scifoni G, Ducci G. Psychopathological assessment of risk of restraint in acute psychiatric patients. J Nerv Ment Dis. 2017;205(6):458–65. doi:10.1097/NMD.0000000000000672. 28319591

[32] Fiorillo A, Giacco D, De Rosa C, Kallert T, Katsakou C, Onchev G, et al. Patient characteristics and symptoms associated with perceived coercion during hospital treatment. Acta Psychiatr Scand. 2012;125(6):460–7. doi:10.1111/j.1600-0447.2011.01809.x. 22176517

[33] Dumais A, Larue C, Drapeau A, Menard G, Giguere Allard M. Prevalence and correlates of seclusion with or without restraint in a Canadian psychiatric hospital: a 2-year retrospective audit. J Psychiatr Ment Health Nurs. 2011;18(5):394–402. doi:10.1111/j.1365-2850.2010.01679.x. 21539684

[34] Andersen K, Nielsen B. Coercion in psychiatry: the importance of extramural factors. Nord J Psychiatry. 2016;70(8):606–10. doi:10.1080/08039488.2016.1190401. 27286476

[35] Lau S, Brackmann N, Mokros A, Habermeyer E. Aims to reduce coercive measures in forensic inpatient treatment: a 9-year observational study. Front Psych. 2020;11:465. doi:10.3389/fpsyt.2020.00465. PMC7267051 32536881

[36] National Institute for Health and Care Excellence (NICE). Violence and aggression: short-term management in mental health, health and community settings. In: NICE guideline, London, United Kingdom: British Psychological Society (UK); 2015. 26180871

[37] Wing JK, Beevor AS, Curtis RH, Park SB, Hadden S, Burns A. Health of the nation outcome scales (HoNOS). Research and development . Br J Psychiatry. 1998;172:11–8. doi:10.1192/bjp.172.1.11. 9534825

[38] Endicott J, Spitzer RL, Fleiss JL, Cohen J. The global assessment scale: a procedure for measuring overall severity of psychiatric disturbance. Arch Gen Psychiatry. 1976;33(6):766–71. doi:10.1001/archpsyc.1976.01770060086012. 938196

[39] World Health Organization (WHO). The ICD-10 classification of mental and behavioural disorders: clinical descriptions and diagnostic guidelines. Geneva: World Health Organization; 1992.

[40] Grace-Martin K. Strategies for choosing the reference category in dummy coding, https://www.theanalysisfactor.com/strategies-dummy-coding/ [accessed 5 December 2022].

[41] Federal Assembly of the Swiss Confederation. Swiss Civil Code. Bern, https://www.admin.ch/opc/en/classified-compilation/19070042/201801010000/210.pdf; 1907 [accessed 5 December 2022].

[42] Kieber-Ospelt I, Theodoridou A, Hoff P, Kawohl W, Seifritz E, Jaeger M. Quality criteria of involuntary psychiatric admissions - before and after the revision of the civil code in Switzerland. BMC Psychiatry. 2016;16:291. doi:10.1186/s12888-016-0998-z. 27520558 PMC4983055

[43] Daffern M, Howells K. Psychiatric inpatient aggression - a review of structural and functional assessment approaches. Aggress Violent Behav. 2002;7(5):477–97. doi:10.1016/S1359-1789(01)00073-8.

[44] Witt K, van Dorn R, Fazel S. Risk factors for violence in psychosis: systematic review and meta-regression analysis of 110 studies. PLoS One. 2013;8(2):e55942. doi:10.1371/journal.pone.0055942. 23418482 PMC3572179

[45] Weltens I, Bak M, Verhagen S, Vandenberk E, Domen P, van Amelsvoort T, et al. Aggression on the psychiatric ward: prevalence and risk factors. A systematic review of the literature. PLoS One. 2021;16(10):e0258346. doi:10.1371/journal.pone.0258346. 34624057 PMC8500453

[46] Burn E, Conneely M, Leverton M, Giacco D. Giving patients choices during involuntary admission: a new intervention. Front Psych. 2019;10:433. doi:10.3389/fpsyt.2019.00433. PMC6620234 31333510

[47] Hirsch S, Steinert T. Measures to avoid coercion in psychiatry and their efficacy. Dtsch Arztebl Int. 2019;116(19):336. doi:10.3238/arztebl.2019.0336. 31288909 PMC6630163

[48] Van de Sande R, Nijman HL, Noorthoorn EO, Wierdsma AI, Hellendoorn E, van der Staak C, et al. Aggression and seclusion on acute psychiatric wards: effect of short-term risk assessment. Br J Psychiatry. 2011;199(6):473–8. doi:10.1192/bjp.bp.111.095141. 22016437

[49] Barbui C, Purgato M, Abdulmalik J, Caldas-de-Almeida JM, Eaton J, Gureje O, et al. Efficacy of interventions to reduce coercive treatment in mental health services: umbrella review of randomised evidence. Br J Psychiatry. 2021;218(4):185–95. doi:10.1192/bjp.2020.144. 32847633

[50] Steinert T. Ethics of coercive treatment and misuse of psychiatry. Psychiatr Serv. 2017;68(3):291–4. doi:10.1176/appi.ps.201600066s. 27691377

[51] Hotzy F, Marty S, Moetteli S, Theodoridou A, Hoff P, Jaeger M. Involuntary admission of psychiatric patients: referring physicians’ perceptions of competence. Int J Soc Psychiatry. 2019;65:580–8. doi:10.1177/0020764019866226. 31379244

[52] Hotzy F, Hengartner MP, Hoff P, Jaeger M, Theodoridou A. Clinical and socio-demographic characteristics associated with involuntary admissions in Switzerland between 2008 and 2016: an observational cohort study before and after implementation of the new legislation. Eur Psychiatry. 2019;59:70–6. doi:10.1016/j.eurpsy.2019.04.004. 31079010

[53] Singh SP, Greenwood N, White S, Churchill R. Ethnicity and the mental health act 1983: systematic review. Br J Psychiatry. 2007;191(2):99–105. doi:10.1192/bjp.bp.106.030346. 17666492

[54] Nørredam M. Migration and health. Danish Med J. 2015;61(4):B5068. 25872539

[55] Bowers L, Douzenis A, Galeazzi GM, Forghieri M, Tsopelas C, Simpson A, et al. Disruptive and dangerous behaviour by patients on acute psychiatric wards in three European centres. Soc Psychiatry Psychiatr Epidemiol. 2005;40(10):822–8. doi:10.1007/s00127-005-0967-1. 16172813

[56] Davis KA, Sudlow CL, Hotopf M. Can mental health diagnoses in administrative data be used for research? A systematic review of the accuracy of routinely collected diagnoses. BMC Psychiatry. 2016;16:263. doi:10.1186/s12888-016-0963-x. 27455845 PMC4960739

[57] Zander E, Wyder L, Holtforth MG, Schnyder U, Hepp U, Stulz N. Validity of routine clinical diagnoses in acute psychiatric inpatients. Psychiatry Res. 2018;259:482–7. doi:10.1016/j.psychres.2017.11.004. 29154169

[58] Egger ST, Weniger G, Prinz S, Vetter S, Mueller M. Health of the nation outcome scales in a psychiatric inpatient setting: assessing clinical change. J Eval Clin Pract. 2015;21(2):236–41. doi:10.1111/jep.12296. 25644710

